# Development of a Translational Assay for Identification and Quantitation of Orthologous Proteins to Aid Drug Development in Preclinical Models

**DOI:** 10.1021/acs.jproteome.5c01164

**Published:** 2026-09-16

**Authors:** Blandine Rougemont, Julie Borgel, Zoe Walker, Irene Zubiri, Ronan O’Cualain, Neda Ali Mohammadi Nafchi, Christopher E. Shaw, Ernestas Sirka, John Isaac, Carlos J. Miranda, Anna Ettorre

**Affiliations:** † Inoviv, Fox Court, 14 Gray’s Inn Road, London WC1X 8HN, U.K.; ‡ AviadoBio Ltd, 20 Water Street, London E14 5GX, U.K.; § Maurice Wohl Clinical Neuroscience Institute, 5 Cutcombe Road, London SE5 9RT, U.K.

**Keywords:** ataxin-2, immunoprecipitation, mass spectrometry, translational science, assay development, drug development

## Abstract

Unequivocal identification and reproducible quantitation of proteins are key components of research and development activity in drug discovery. In some cases, the target protein might be a protein highly conserved across species; antibody-based assays are not specific enough to discriminate between orthologues, thus forcing drug developers to use multiple analytical platforms across preclinical models. Here, we present a modular mass spectrometry-based assay for the analysis of orthologous proteins across species in the context of translational science using ataxin-2 (ATXN2) as an example. Instead of relying on recombinant proteins, we applied rational peptide design to target human, mouse, human/pig/non-human primates (NHP), or pan-species regions of the full-length protein. Specificity and sensitivity of the method were proven in wild-type and transgenic models. Dose–response upon treatment with different doses of an adeno-associated virus (AAV)-delivering microRNA (miRNA) targeting ATXN2 showed that knock-down at the transcript level was consistent with downregulation at the protein level, confirming the assay suitability for target engagement evaluation. Furthermore, the data presented here indicate that this method is readily transferable to minipigs, ensuring analytical continuity across preclinical studies. More broadly, this work provides a translational blueprint that can be adapted to other orthologues, reducing cost and timeline in drug development.

## Graphical abstract

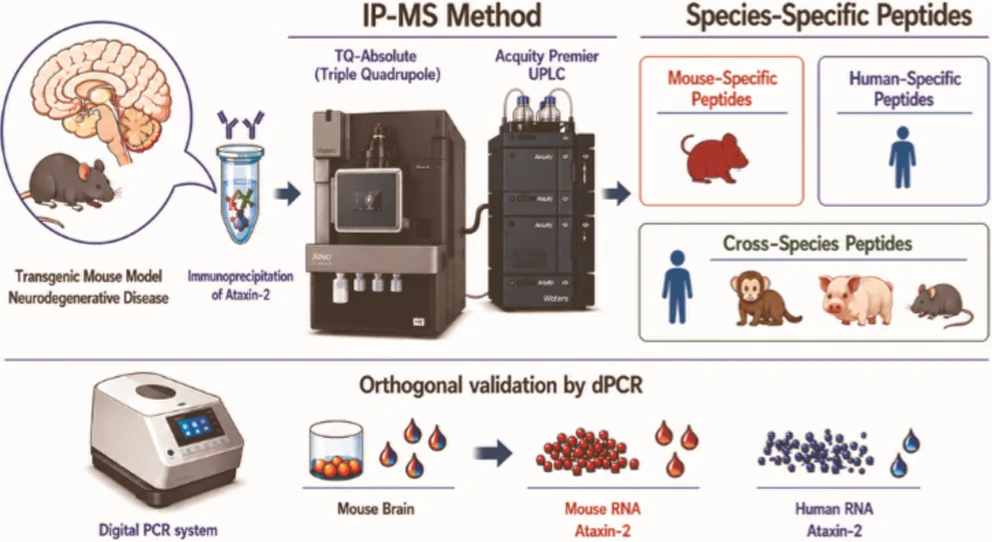

## Introduction

Translational research for drug discovery and development has changed in recent years with increasing importance of biomarker-driven evidence of target engagement and efficacy.^1^ Biomarkers are identified earlier on at preclinical stage, characterized in disease animal models with the aim of a seamless translation to the clinical setting.^2^

Different animal species are used in preclinical studies, depending on the type of study, which complicates the translational aspect of assay development even further.

Assay development and validation can be expensive as well as time-consuming.^3^ An unsuitable, not fit-for-purpose, or poorly performing assay will delay pipeline progression. For this reason, translational assay development requires a multidisciplinary approach, involving different techniques to enable cross-checking of data generated with another assay using a completely different technique.

Here, we present an example of a translational approach to develop an assay that will deliver the ability to discriminate between proteins with extremely high homology between species while also accounting for the possibility of being used to analyze tissue samples from different species in preclinical studies, in particular toxicological studies.

We have a long-standing interest in finding new disease-modifying therapies for motor neuron disorders, in particular, for amyotrophic lateral sclerosis (ALS). One of the most promising targets for ALS therapies is a reduction in ataxin-2 (ATXN2) expression, which represents the embodiment of all translational challenges described above for orthologous proteins. In this study, we present the strategy employed by our team to develop a fully translational cross-species assay for the ATXN2.

ATXN2 is a glutamine-rich (polyQ) protein, with a low structural complexity, containing on average 22 glutamine repeats.^4^ Although its function is not fully elucidated, ATXN2 is involved in RNA and cellular metabolism, circadian rhythms, cytoskeleton reorganization, and regulation of Ca^2+^ homeostasis.^5^ Genomic expansion of >32 trinucleotide CAG repeats encoding polyglutamine (CAG polyQ) repeats in ATXN2 gives rise to Spinocerebellar Ataxia Type 2 (SCA2), an autosomal dominant neurodegenerative disorder primarily affecting the cerebellum, brainstem, and spinal cord. Intermediate expansions of 29–32 CAG (polyQ) repeats in ATXN2 are a risk factor for ALS and frontotemporal dementia (FTD).^6^ Multiple oxidative and osmotic stressors provoke the formation of cytoplasmic stress granules which is facilitated by ATXN2. Knockout or knock-down of ATXN2 has been shown to suppress toxicity due to the cytoplasmic accumulation of transactive response DNA-binding protein 43 kDa (TDP-43) in multiple cellular and animal models of ALS or tauopathies.^6–9^ Small molecules like integrated stress response inhibitor (ISRIB), stress granuli inhibitors, or ATXN2 antisense oligonucleotides (ASO) suppress neurodegeneration in a C9-ALS-induced pluripotent stem cell (iPSC)-derived neurons (iPSNs), further supporting critical roles for SG assembly and nucleocytoplasmic transport disruption in the pathogenesis of these diseases.^7^ A single administration of ATXN2-ASO at birth in a TDP-43 ALS mouse model showed sustained and significant reductions in ATXN2 mRNA levels, prolonged survival, and improved motor function.^10^ Taken together, these studies support ATXN2 as a potential drug target for neurodegenerative disorders.

Mice transgenic for the human *ATXN2* bacterial artificial chromosome (BAC) transgene with an expanded CAG repeat (BAC-Q72) developed a slowly progressive cellular and motor phenotype and are particularly useful to test new drugs that interfere with polyQ-ATXN2-induced neurotoxicity.^11^ The drawback is that these mice express endogenous mouse *Atxn2*, and like many orthologues, ATXN2 proteins in different species share a high degree of homology.

Complicating the matter further, commercially available antibodies are not species-specific. Indeed, the availability of an assay able to discriminate and confidently measure mouse *vs* human, pig, and nonhuman primate (NHP) proteins is crucial in preclinical studies and for drug development overall.

In this study, we developed an immunoprecipitation (IP) coupled to mass-spectrometry (MS) method to quantify the relative expression of human *vs* mouse ATXN2 proteins in tissue lysates. The IP-MS method is sensitive, reproducible, and specific, as demonstrated using brain tissue from BAC-Q72 mice treated with different doses of an AAV-miR-ATXN2 gene therapy product designed to selectively knock down both mouse and human ATXN2, named AAV9-miR-ATXN2. This IP-MS method has been orthogonally tested using reverse transcription digital PCR (RT-dPCR), demonstrating that changes at the protein level in the expression of species-specific ATXN2 can be measured and correlate with transcript expression levels.

The strategy devised in this study differs slightly from that of a classical proteomics workflow.

The identification and quantification of mouse *vs* human ATXN2 protein was obtained by selecting specific peptides to target human, mouse, human/pig/NHP, or pan-species regions of the full-length protein. Cross-reactive human/pig/NHP peptides were chosen for use in species suitable for good laboratory practice (GLP) toxicology and other translational studies. To ensure assay selectivity and specificity, we used lysates from multiple tissues and knockout models. This strategy, leveraging biologically relevant samples from different transgenic animals, provides a fit-for-purpose assay through an efficient and pragmatic experimental design that maintains work-flow continuity while reducing cost and timeline in preclinical drug development.

## Materials and Methods

### *Ex Vivo* Tissue Sample Collection for Assay Development

For assay development purposes, brains, spinal cord, liver, muscles, and blood samples were collected from BAC-Q72 ^11^ crosses and wild-type (WT, C57BL/6J strain) mice at King’s College Facility in London (UK); brains, spinal cord (lumbar and thoracic sections), liver, muscles, blood, and cerebrospinal fluid (CSF) were collected from Yucatan minipig at Neurgain facility in San Diego (US). All animals were euthanized, tissues perfused with saline, kept refrigerated during the procedure, subsequently snap-frozen, and stored at -80° C. *Atxn2* knockout (KO) brain samples were kindly donated by Stefan Pulst, University of Utah.^12^ Brain samples from *Atxn2* KO and heterozygous mice were collected from two 7 week-old mice (one heterozygous mouse and one homozygote mouse) and two 20 week-old mice (one heterozygous mouse and one homozygote mouse). The average tissue weight was 50 mg.

### Tissue Lysis

Tissue samples were lysed in 2 mL of IP Lysis buffer (87788, Thermo Scientific Pierce) supplemented with protease inhibitor cocktails (78425, Thermo Scientific) and mechanically lysed on a Precellys using a Precellys Evolution Homogenizer (Bertin, 3 cycles of 30 s at 5000 rpm, with 30 s rest using cooler in between each cycle).

Total protein recovery was measured using the Pierce 600 nm Protein Assay (Thermo Scientific Pierce, 22662) and ranged from 1 to 2 mg/mL for mouse tissue lysates and from 7 to 10 mg/mL for minipig tissue lysates.

### ATXN2 ImmunoPrecipitation

An initial assessment to identify the best antibody (Ab) cocktails for immunoprecipitation (IP) was performed using four antibodies (Abs). All four Abs were cited in previously published studies and raised against known immunogens: rabbit polyclonal Ab PA5-54183 (Invitrogen; RRID: AB_2638321); rabbit polyclonal Ab PA5-40131 (Invitrogen; RRID: AB_2605838); mouse monoclonal Ab BD-611378 (clone 22, BD Bioscience Transduction Laboratories; RRID: AB_398900); rabbit polyclonal Ab 21776-1-AP (Proteintech; RRID: AB_10858483).

This initial assessment was performed by loading 2 μg of each individual antibody onto Protein-G magnetic Dynabeads (Invitrogen, 10004D). The IP step was performed exactly as described below for the automated protocol.

For all further experiments, an automated system (Assay Map Bravo, AMB, Agilent) was used for the IP of ATXN2 in tissue lysates and biofluids.

A mixture containing 3.5 μg of each of the two *anti*-ATXN2 antibodies selected (Invitrogen PA5-54183 and Proteintech, 21776–1-AP) was loaded on Protein-G cartridges (Agilent, PG-W G5496–60008) using the “Immobilization Protocol” standard parameters on the AMB, loading 50 μL of mixture at 5 μL/min.

Tissue lysates were diluted 1:1 v/v in phosphate-buffered saline (PBS) before being loaded onto the antibody bound cartridges, loading the equivalent of approximately 300 μg of total protein. The same procedure was followed for biofluids, loading up to 1 mL of CSF and 250 μL of plasma.

IP of ATXN2 proteins was performed using the “Affinity Purification Protocol” standard parameters on the AMB, loading the diluted lysate at 3.5 μL/min, performing a PBS-Tween and a PBS wash before eluting the target in 50 μL of glycine 0.1 M.

### Sample Preparation for LC–MS/MS

The enzymatic digestion was done using the AMB “In-Solution Digestion Single Plate Protocol” application. Briefly, the entire volume of the IP eluate was diluted in triethylammonium bicarbonate buffer (TEAB 50 mM), Rapigest 0.1% (Waters, 186001861), reduced with dithiothreitol (DTT 100 mM), alkylated using iodoacetamide (IAM 200 mM), before being digested with trypsin overnight.

The resulting peptide mixture was dried down under nitrogen flow and resuspended in 50 μL of 0.1% formic acid (FA) in LC–MS water.

### Multiple Reaction Monitoring (MRM) Proteomic Analysis and Quantification

Peptide samples were analyzed on a Xevo TQ Absolute mass spectrometer (Waters) coupled to an ACQUITY UPLC I-Class (Waters) liquid chromatography system. Samples were separated on an ACQUITY UPLC column of 15 cm length and 1 mm internal diameter (HSS-T3, C18, 100 Å, 1.8 μm particle size) maintained at 60 °C, using a linear gradient of 3%–35% acetonitrile at a flow rate of 100 μL/min over 15 min. The eluent was analyzed in a scheduled MRM mode, following electrospray ionization in the positive mode (1 kV spray voltage, 300 °C desolvation temperature). The optimized MRM method monitored, in a scheduled acquisition, 98 transitions corresponding to 16 peptides and their labeled internal standard counterparts.

Stable isotope-labeled internal standard peptides (heavy) were labeled on their C-terminal residue, and both light and heavy peptides were synthesized for method development and optimization (Pepmic Co., Suzhou, China).

### Data Analysis

The chromatogram data were extracted using Skyline software^13^ (MacCoss Lab, University of Washington), manually inspected, and exported for further analysis. Relative quantification data are given as a ratio to labeled internal standard and normalized to tissue weight. Peptide graphs, comparison, and correlation plots were generated using our in-house R code.

Protein concentration comparisons across categorical variables were performed using the Wilcoxon rank-sum test. Correlations between protein concentrations and reverse transcription digital polymerase chain reaction (RT-dPCR) or quantitative polymerase chain reaction (qPCR) data, as well as other continuous variables, were assessed using Spearman’s rank correlation. All statistical tests were two-sided, with significance defined as *p* < 0.05. Analyses were conducted using a bespoke R script.

### *In Vivo* Mouse Study Using AAV9-miR-ATXN2

Neonatal (P0–P2) mice from BAC-Q72, expressing human Ataxin-2, were treated *via* intracerebroventricular (ICV) injections with AAV9-miR-ATXN2 (Dose1: 1.56 × 10^11^; Dose 2: 5.19 × 10^10^; Dose 3: 1.73 × 10^10^, total vector particles injected per mouse pup), AAV9-miR-Neg (1.50 × 10^11^ total vector particles injected per mouse pup), or vehicle (Dulbecco’s phosphate-buffered saline, DPBS, Gibco, Invitrogen). A naïve group was also included as an untreated uninjected control. At 8 weeks post injection, mice were euthanized, and tissues were collected for bioanalyses. DNA and RNA were extracted from the left hemisphere cortex to measure the presence of vector genome by qPCR and *ATXN2* and *Atxn2* by RT-dPCR, respectively. The right hemisphere cortex was snap frozen and used for protein analysis.

### Tissue Processing for RT-dPCR Analysis

All tissues were stored at -80 °C in homogenization tubes (MP Biomedicals). Prior to processing, tissues for DNA and RNA extraction were suspended in 500 μL (cortex) or 700 μL (liver) cold RLT Buffer with 40 mM of DTT (Qiagen). A 5 mm diameter stainless steel bead (Qiagen) was added to each tube. The tubes were placed in a cooled Fastprep machine adaptor. Cortex and spinal cord samples were homogenized at a speed of 6.0 m/s for 40 s (×2 runs) in a Fastprep-24 5G homogenizer (MP Biomedicals). Liver biopsies were homogenized as above for ×3 runs.

All DNA and RNA extraction samples were centrifuged at 9,400*g* for 2 min at 4 °C. After, tubes were vortexed briefly and placed on wet ice and aliquoted for RNA (∼200 μL) and gDNA (∼200 μL) extractions. Any spare homogenate was stored at -80° C.

### Total RNA Extraction

Homogenates were aliquoted in 2 mL tubes for downstream RNA extraction with the QIAsymphony and RNA kit (Qiagen), using the miRNA_CT_400_V8 protocol. The aliquoted volume was diluted with 200 μL of RLT buffer +100 μL chloroform, vortexed thoroughly, then centrifuged at 13,000*g* at 4 °C for 3 min. RNA was eluted in 50 μL of nuclease free water.

### RNA Quantification

The concentration and purity of DNA and RNA were determined by UV spectrophotometry using a NanoDrop Eight Spectrophotometer (Labtech). Following extraction, 1.1 μL of gDNA or RNA was loaded onto the optical pedestal and absorbance of samples were read at 230, 260, and 280 nm (A230, A260, and A280). For RNA samples, nuclease free water was used as a blank, and for DNA samples, buffer ATE (Qiagen) was used.

### Quantification of Human *ATXN2*, Mouse, and Minipig *Atxn2* by RT-dPCR

Reverse transcription reactions were carried out on using the LunaScript RT SuperMix (NEB) master mix (containing random hexamers and poly dT primers). Reaction mixtures were prepared, containing 200 ng of RNA, 4 μL of Supermix, and water up to 20 μL (total reaction volume). Sample RT reactions were prepared with the same conditions in a separate RT reaction setup. Thermo cycling was carried out as follows in a ProFlex PCR System (Applied Biosystems): 2 min at 25 °C, 10 min at 55 °C, 1 min at 95 °C. After cycling, the cDNA samples were diluted 1 in 200 in nuclease free water.

RNA levels were analyzed in a Digital PCR (dPCR) system (Qiagen), in 24-well 26,000 partitions (26K) plates. Samples were amplified in a final volume of 40 μL, contain 4× Probe PCR Master Mix (Qiagen), 5 μL of diluted cDNA, 0.3 μM of each forward and reverse primer, and 0.375 μM of each probe. After partitioning, PCR reactions were performed under the following thermal cycler conditions: 2 min at 95 °C, followed by 40 cycles of 15 s at 95 °C, 30 s at 60 °C. Imaging was carried out for 300 ms, with a gain of 6 in the green and yellow channels.

The analysis was performed by determining the Copy/μg RNA for the target (*ATXN2* or *Atxn2*) and the housekeeping gene (*Gapdh*) considering 0.25 ng of RNA in each dPCR well. The target expression was then normalized by the *Gapdh* expression. The normalized values for each animal were then expressed as a percentage of the averaged normalized values of the naïve group. All primers and probes were purchased from Integrated DNA Technologies (IDT, Coralville, Iowa, US).

*Gapdh*: forward primer, CATCCACTGGTGCTGCCAAG; reverse primer, TCAGATGCCTGCTTCACCACC; probe, CCCAGAGCTGAACGGGAAGCTCACTGGCAT; fluorophore, HEX.

*ATXN2*: forward primer: AGGGGCACAGCATAAACACT; reverse primer: ATCTTGATGGCATGGAGCCC; probe: AGTGGGAGACAGAATTCACCGCGTAT; fluorophore: FAM/ZEN.

*Atxn2*: forward primer: ACCAAGCCAAGACATATAGAGC; reverse primer: CGTACTGAGTGGAGTAAGCGG; probe: CACCATGATGCACCCAGCC; fluorophore: FAM/ZEN.

## Results

### IP-MS Method Development and Optimization

#### Peptide Selection, Identification, and Optimization

A total of 28 peptides were designed to quantify ATXN2 protein: 7 peptides specific for mouse; 7 peptides specific for human; 7 peptides common to human, pig, and NHP; and 7 peptides common to all four species (human, mouse, pig, and NHP). The 7 peptides specific for human ATXN2 were to be tested in human samples and only in case of a poor performance of the selected mouse/human/pig/NHP peptides or cross-reactive human/pig/NHP peptides. This was decided in order to prioritize the translatability of the assay across multiple phases of the drug development life cycle. A complete list of the peptides can be found in Supporting Information Table S1.

The amino acid sequences of ATXN2 proteins for the different species used for sequence alignment during the peptide design stage were taken from UniProt database: Q99700 (*H. sapiens*, human ATXN2); O70305 (*M. musculus*, murine ATXN2); F1RMZ0 (*S. scrofa*, pig ATXN2), and H9F864 (*M. mulatta*, NHP ATXN2) (Supporting Information Figure S1).

Peptide tuning was performed to clearly define solubility, the optimal precursor ion; fragment ions, voltage, and retention time for each peptide, as well as optimal profile on the LC–MS system using IntelliStart software (Waters).

For each selected peptide reported in Supporting Information Table S1A, a heavy labeled internal standard peptide was synthesized. To monitor digestion performance, each internal standard peptide included a 4–6 amino acid tryptic tag designed to replicate the sequence of the species-specific ATXN2 proteins. This design ensures that the standards are cleaved in a manner comparable to the native peptide, thus permitting quantitative evaluation of enzymatic efficiency and consistency throughout the workflow (Supporting Information Table S1B). An example of chromatogram for the peptides tested can be found in Supporting Information Table S1C.

#### Antibody Cocktail Optimization for the IP Step

The strategy deployed in this study differs slightly from that of a classical proteomics workflow (Figure 1). Recombinant proteins are often used as standards in proteomic workflows; however, this approach was not feasible for ATXN2. ATXN2 is a large 140 kDa protein with allelic variants and intrinsically disordered regions, making the proper folding of a full-length recombinant protein technically challenging. Moreover, to our knowledge, full-length recombinant ATXN2 proteins are not commercially available for any of the species relevant to this study (human, mouse, pig, or NHP), and only partial fragments can be sourced. Although internal protein synthesis capabilities could have enabled production of such fragments, their use would not have provided an appropriate standard as the absence of complete sequence coverage, particularly across regions targeted by our peptide design, would have introduced bias as well as increased cost and significantly extend the project timeline. Instead, to ensure assay selectivity and specificity, we employed lysates from multiple tissue types and knockout models, complemented by light and heavy peptides with flanking amino acid regions. The reproducibility of the workflow on tissue samples was further confirmed by repeated extractions of the tissue samples from different genetic backgrounds. This strategy, leveraging biologically relevant samples from different transgenic animals collected by our collaborators during *in vivo* studies run at any time in their laboratories, provides a more representative and robust standard than partial recombinant proteins and aligns with our objective of developing a fit-for-purpose assay through an efficient and pragmatic experimental design.

**Figure 1:**
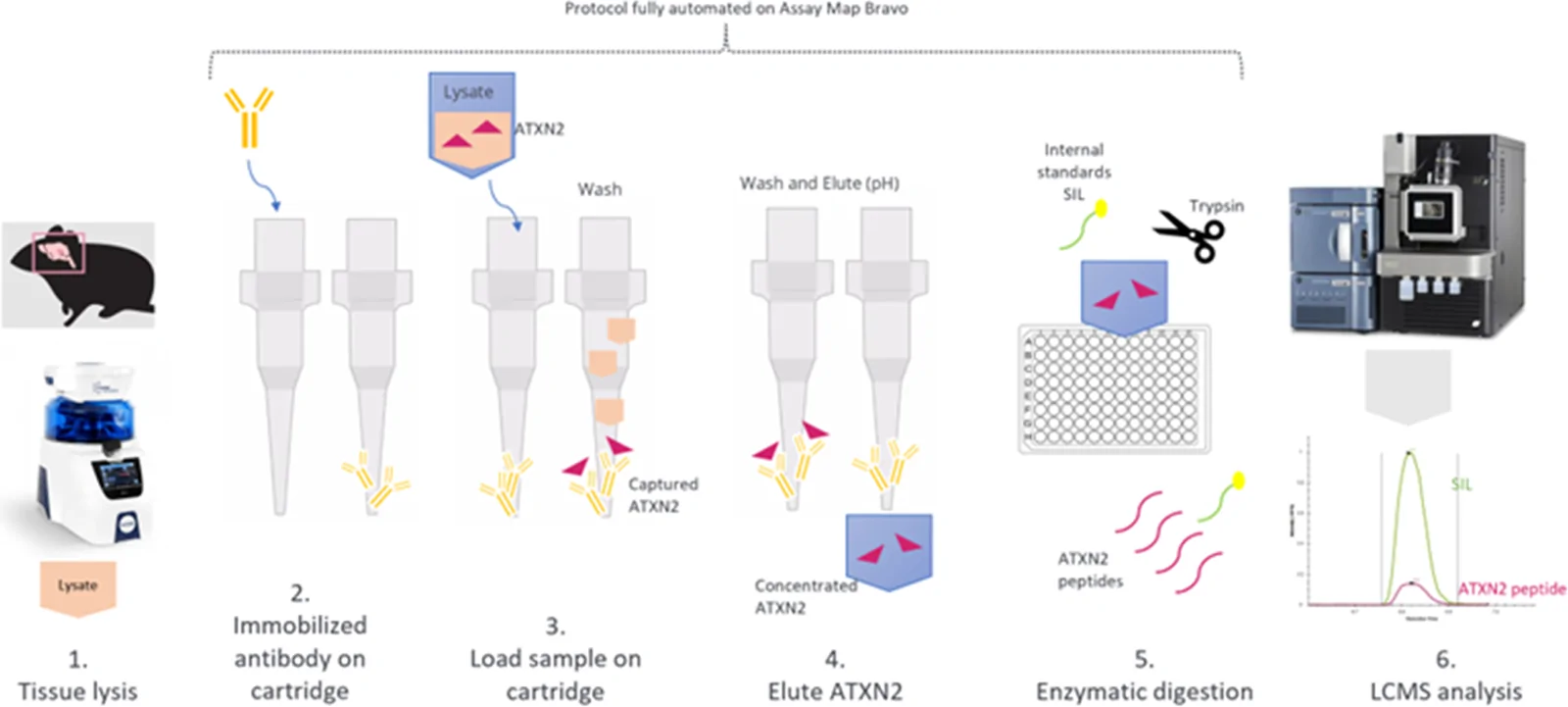
Workflow of the developed IP-MS assay for ATXN2 proteins. Schematic workflow for the IP-MS assay developed in this study to identify species-specific peptides for ATXN2 orthologous proteins. The protocol has been fully optimized for automated workstations to reduce time and manual error during the entire process.

The next step was to identify the optimal antibody cocktail to immunoprecipitate mouse and human ATXN2 proteins in the sample mix. Four antibodies (Abs), cited in previously published studies and with known immunogens, were tested: rabbit polyclonal Ab PA5-54183; rabbit polyclonal Ab PA5-40131; mouse monoclonal Ab BD-611378; and rabbit polyclonal Ab 21776-1-AP. Each antibody was immobilized on magnetic beads, then incubated with liver homogenate or plasma from one WT and one BAC-Q72 mouse, enzymatically digested with trypsin and analyzed using LC–MS (Figure 1).

With this experiment, the ability of different antibodies to bind and selectively precipitate ATXN2 protein was tested by tracking the sequence of peptides specific for mouse ATXN2 or those common across all four species (see peptide list and sequence in Supporting Information Table S2).

With PA5-40131 Ab, neither mouse nor cross-reactive peptides could be identified by LC–MS (Figure 2A). With BD-611378, four cross-reactive peptides and two mouse specific peptides could be detected in liver homogenate of both WT and BAC-Q72 mice but not plasma, thus suggesting that this antibody recognizes specific tissue variants of ATXN2 proteins (Figure 2A). Both PA5-54183 and 21776-1-AP detected five cross-reactive and three mouse specific peptides in liver samples of WT and BAC-Q72 animals (Figure 2A). Interestingly, plasma samples showed differences in the peptides identified upon IP with the two antibodies, probably due to the epitope recognized. With PA5-54183 Ab two cross-reactive and two mouse, and two cross-reactive and three mouse peptides were identified in BAC-Q72 and WT mice, respectively (Figure 2A). With 21776-1-AP Ab, one cross-reactive and two mouse, and two common and three mouse peptides were identified in BAC-Q72 and WT mice, respectively (Figure 2A).

**Figure 2:**
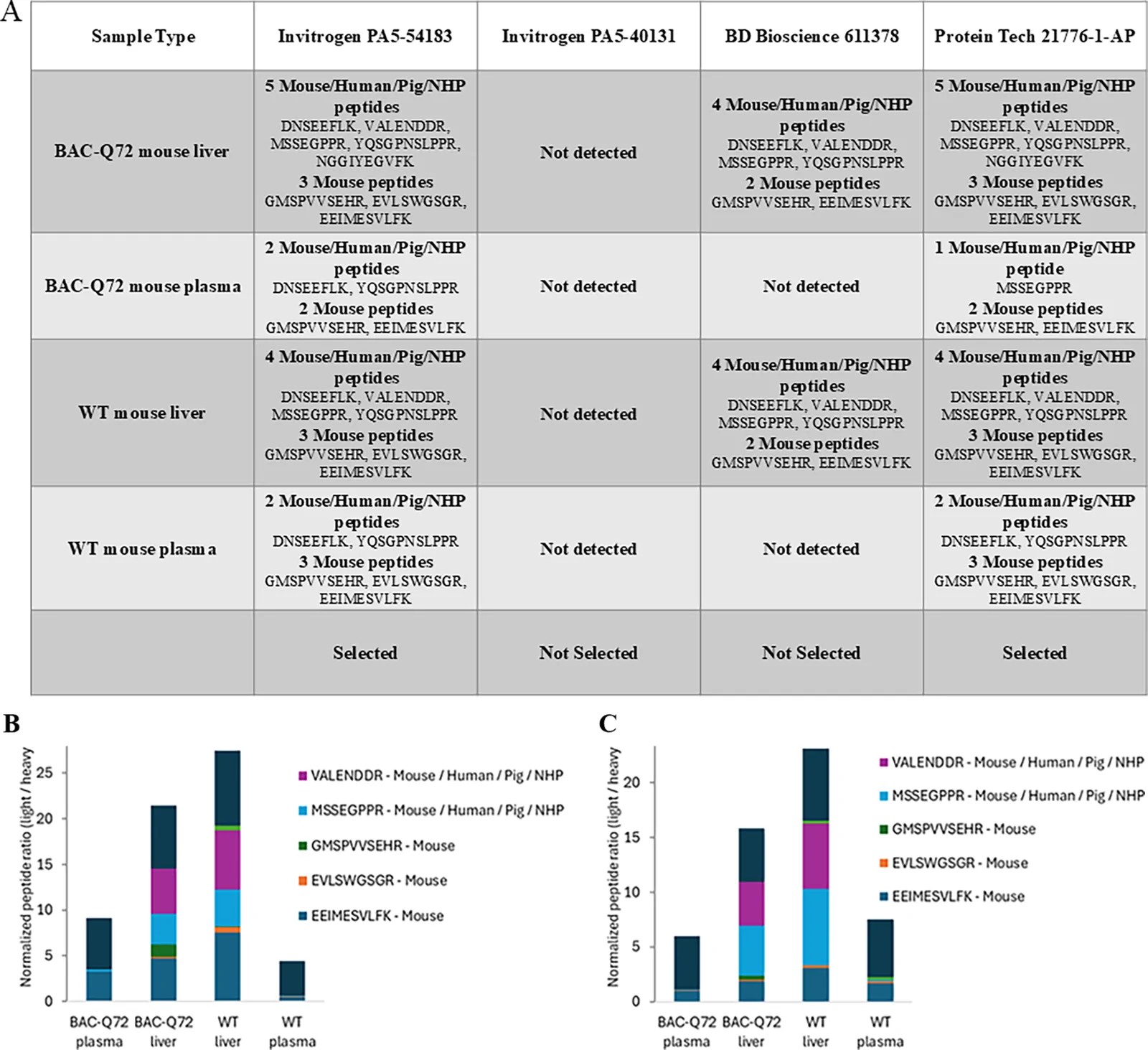
Evaluation of commercially available antibodies suitable for use in IP-MS assay development. Four different commercially available antibodies were tested in pull-down experiments for their ability to bind to human and mouse ATXN2 from two complex matrices and identify mouse-specific and cross-reactive mouse/human/pig/NHP peptides. Liver and plasma from WT and BAC-Q72 mice were used in this experiment to account for tissue-specific variants of ATXN2 protein. (A) Results using single antibody pull-down test and peptides identified are summarized in the table reported in panel (A). Peptide signal for mouse-specific and crosss-reactive mouse/human/pig/NHP peptides recorded with the use of PA5-54183 (B) and 21776-1-AP (C) antibodies.

PA5-54183 and 21776-1-AP Abs were chosen to be used as part of the antibody cocktail mixture to immunoprecipitate both mouse and human ATXN2 proteins in future experiments, thanks to their broader coverage in terms of peptides identified in two different matrices (Figure 2B–C).

Therefore, the workflow outlined in Figure 1 was followed using the selected antibodies PA5-54183 and 21776-1-AP for the rest of the experiments described in this study.

#### Identification and Quantification of ATXN2 Peptides in Mouse and Minipig Tissues

The next step was to verify whether the peptides selected were clearly and unambiguously identified in neuronal (brain and spinal cord) *vs* non-neuronal (liver) tissues dissected from WT­(C57BL/6J) and BAC-Q72xWT­(C57BL/6J) mice and whether there was a difference in any of the peptides recognizing specific tissue variants of ATXN2 protein. As predicted, only peptides common to all four species and mouse-specific peptides were identified in the WT mouse samples; likewise, all three groups of peptides were identified in the transgenic BAC-Q72 mouse samples as these mice overexpressed human ATXN2 as well as still expressed endogenous mouse ATXN2 protein (Figure 3A). Special consideration was given to specific areas of the brain and the spinal cord; mouse-specific peptides showed a comparable level of expression and similar pattern across the dorsal or ventral area of the brain, upper and lower spinal cord between WT and BAC-Q72 animals, as such the different areas were combined together into brain (B) and spinal cord (SP) respectively (Figure 3A). Mouse and human/pig/NHP peptides showed comparable expression levels and similar patterns across different areas of brain and spinal cord in BAC-Q72 mice, thus suggesting that expression of human *vs* mouse BAC-Q72 in transgenic animals is similar (Figure 3A).

**Figure 3:**
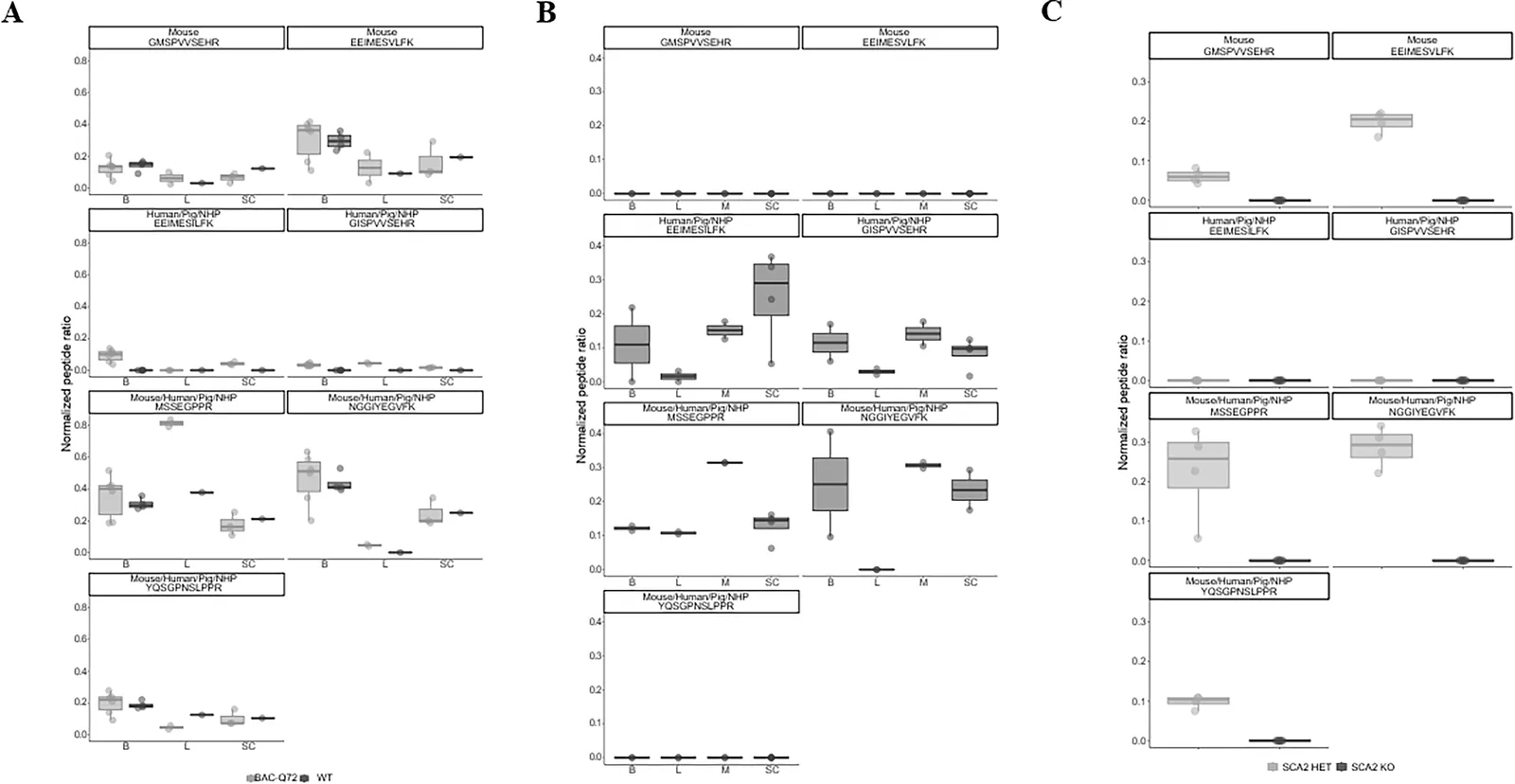
Identification and quantification of ATXN2 peptides in mouse and minipig tissues. Mouse-specific, human/pig/NHP and cross-species mouse/human/pig/NHP peptides designed against ATXN2 proteins were identified in brain (B) and spinal cord (SC) as well as in liver (L) specimens of BAC-Q72 and WT mice (A). The same peptides were also tested in pig brain (B), liver (L), muscle (M), and spinal cord (SC) samples to prove species-specificity of the mouse-specific peptides *vs* species cross-reactivity of the human/pig/NHP and cross-species mouse/human/pig/NHP peptides (B). Selectivity and specificity of the three sets of peptides were further tested in brain samples from SCA2 HET and SCA2 KO mice expressing or null for mouse *Atxn2* (C).

Cross-reactive mouse/human/pig/NHP peptides follow the same pattern but show a higher signal in BAC-Q72 mice, possibly due to the combined expression of both mouse and human ATXN2 proteins.

To prove the potential translatability of the assay to other preclinical species, we assessed whether cross-species human/pig/NHP and mouse/human/pig/NHP peptides could also recognize ATXN2 protein in minipig tissue samples. Brain, spinal cord (upper and lower sections, combined together in Figure 3B), liver, and muscle tissue biopsies were tested using the same peptides previously tested in mouse specimens. To confirm the species specificity of the assay, no mouse-specific peptides were identified in those samples, only human/pig/NHP peptides and two out of three mouse/human/pig/NHP peptides.

Interestingly, one peptide (EEIMESILFK) from the human/pig/NHP group and one peptide (NGGIYEGVFK) from the mouse/human/pig/NHP group showed a higher recovery for ATXN2 protein in neuronal tissues. Of note, only one peptide (MSSEGPPR) from the mouse/human/pig/NHP group showed a preferential recognition of ATXN2 in muscle tissue (Figure 3B).

The difference in peptide recovered from muscular *vs* brain tissue can be explained with tissue-specific isoforms of ATXN2 (UniProt entry: Q99700), or a conformation-dependent phenomenon, as ATXN2 presents different disordered regions, upon interaction with other protein in the lysate. These results will need further investigation, if the assay will be used to differentiate tissue isoforms in disease setting and pharmacological studies.

#### Assessment of Selectivity and Specificity of the Methodology

Brain samples from SCA2 (ATXN2) KO and heterozygous mice were collected from two 7 week-old mice (one heterozygous mouse and one homozygote mouse) and two 20 week-old mice (one heterozygous mouse and one homozygote mouse).

The same seven peptides belonging to the three groups tested in the experiments described above were also tested for specificity in the KO and HET samples from the SCA2 mice. None of the seven peptides were identified in the SCA2 (ATXN2) KO samples as those mice express neither the mouse nor human ATXN2 protein (Figure 3C). Moreover, in the HET mice, only mouse-specific and mouse/human/pig/NHP cross-reactive peptides were identified, but none of the peptides belonged to the human/pig/NHP group (Figure 3C).

There did not seem to be an age-related difference in the amount or type of peptides identified, or lack thereof. Albeit on a small number of samples, this experiment confirmed that the designed peptides are both specific and selective, and the assay developed is fit for purpose.

#### Linearity and Reproducibility

Assessment of linearity and reproducibility was performed to further characterize the IP-MS assay and confirm its suitability as a fit-for-purpose method for the relative quantification of ATXN2 orthologous proteins in preclinical studies (Figure 4). Custom-made synthetic light and heavy peptides were spiked into a surrogate matrix (0.1% bovine serum albumin, BSA) to generate calibration curves spanning 100 ng/mL to 32 pg/mL. Each concentration point was prepared in triplicate. Linearity was evaluated using linear regression, and acceptance criteria were defined as ≤20% coefficient of variation (CV) for precision and ≤20% bias for accuracy across all standards.

**Figure 4:**
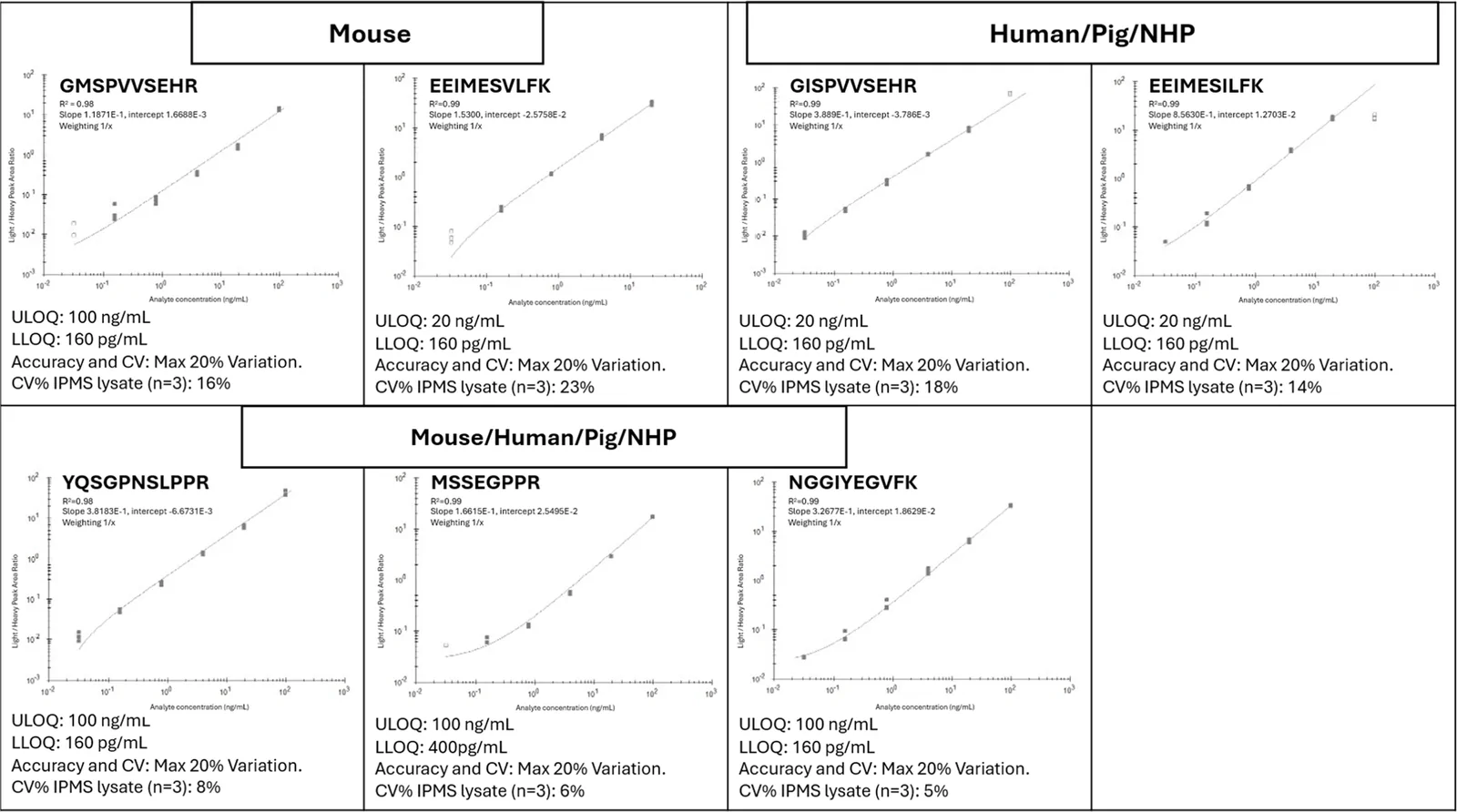
Linearity and reproducibility assessment for the selected peptides. Assessment of linearity and reproducibility for mouse-specific, human/pig/NHP, and cross-species mouse/human/pig/NHP peptides. Abbreviations used in the figure: (ULOQ, upper limit of quantitation; LLOQ, lower limit of quantitation; CV, coefficient of variation; *R* ^2^, coefficient of determination).

With the exception of MSSEGPPR, all peptides met these criteria down to a lower limit of quantification (LLOQ) of 160 pg/mL, demonstrating strong linear behavior and high analytical sensitivity (Figure 4). The reduced performance of MSSEGPPR might be due to the intrinsic physicochemical properties of the peptides.

To evaluate reproducibility across the full workflow, a pooled BAC-Q72 cortex lysate was processed in triplicate using the optimized IP-MS protocol. Peptides specific to mouse ATXN2 and those unique to human/pig/NHP orthologues were successfully quantified and showed CVs below the 20% limit (with CVs of 14-18%), with only one peptide (EEIMESVLFK) that showed CV just above the 20% fixed limit (CVs of 23%). Peptides conserved across all species showed markedly higher reproducibility, with CVs of 5-8%. The higher CVs registered for peptides specific to mouse ATXN2 are likely to reflect a lower protein abundance and a greater variability in tissue-derived samples.

CVs slightly above 20% of the EEIMESVLFK peptide were considered acceptable for tissue-derived samples due to inherent biological heterogeneity. As its higher CVs did not seem to impact the overall quantitative interpretation, it was included in the panel of peptides measured in further studies.

Collectively, the assay demonstrates robust linearity, high sensitivity, and reproducible performance across both synthetic standards and biological matrices. These analytical characteristics support the use of the developed IP-MS assay for the relative quantification of mouse and human ATXN2 orthologues in murine tissues within preclinical studies.

#### ATXN2 Downregulation at mRNA and Protein Levels in Cortex of Mice Treated with AAV9-miR-ATXN2

The next and final experiment described below was designed to confirm that the IP-MS assay developed so far was fit-for-purpose and sensitive enough to be used in preclinical samples to measure ATXN2 protein downregulation following a pharmacological treatment targeting ATXN2.

AAV9-miR-ATXN2 is an AAV9 vector expressing a miRNA targeting ATXN2. BAC-Q72 P0-P2 litter mice were injected intracerebroventricularly (ICV) with three doses (expressed as total vector particles injected per mouse pup) of AAV9-miR-ATXN2: Dose 1: 1.56 × 10^11^; Dose 2: 5.19 × 10^10^; and Dose 3: 1.73 × 10^10^. A nonrelevant construct (AAV9-miR-Neg) was used as negative control at a dose approximately matching Dose 1 of AAV9-miR-ATXN2 to account for nonspecific effects on ATXN2 mRNA expression. A group of naïve mice were added to the treated cohorts to rule out any potential procedural-related issues which might have affected the regulation of ATXN2 proteins and were independent from the treatment. At 8 weeks post injection, mice were euthanized, and tissues were collected for bioanalyses. The right-brain hemisphere was used for protein analysis, and left-brain hemisphere was used for both vector genome determination and mRNA analysis of human and mouse mRNA transcripts. The samples for DNA/RNA and protein analysis were processed independently in two different facilities.

AAV9-miR-ATXN2 was designed to target both human and mouse *ATXN2* transcripts. To prove target engagement of the AAV9 vector upon ICV delivery in the mouse brain, RT-dPCR was employed to quantify the amount of human and mouse *ATXN2* transcript. The RT-dPCR technique guaranteed a higher sensitivity than RT-qPCR to appreciate changes in the mRNA level of ATXN2, whose expression at the cellular level is not particularly high.

Reduction of *ATXN2* mRNA did not only show a dose–response but was also statistically significant for all three doses tested (Figure 5A, left-hand side panel). Reduction of *Atxn2* mRNA was statistically significant only for the highest dose tested (Figure 5A, right-hand side panel).

**Figure 5:**
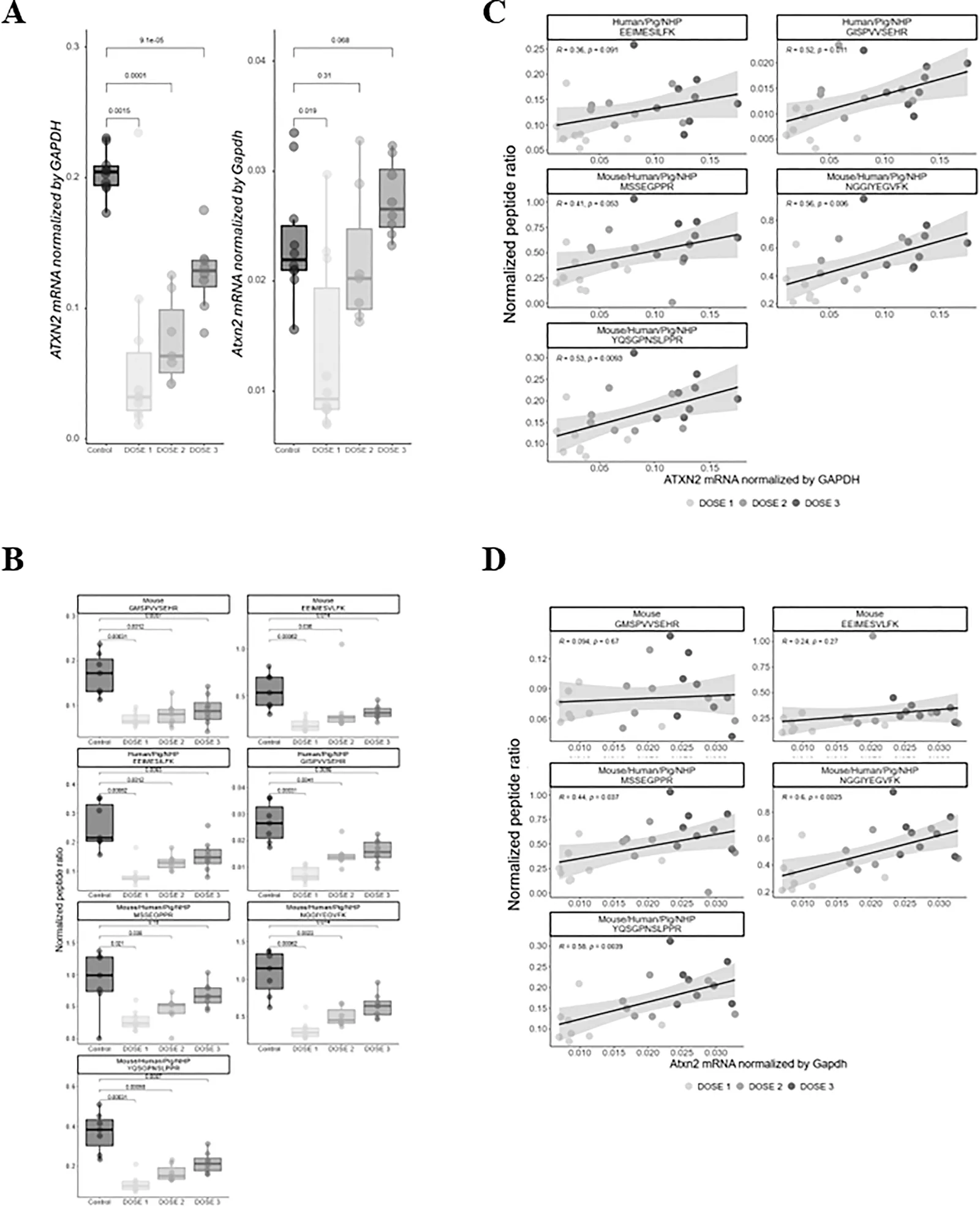
Assay fitness evaluation of the IP-MS method developed: ability to discriminate between mouse and human ATXN2 in BAC-Q72 mice. IP-MS method suitability and performance were assessed in the Proof of Mechanism (PoM) study from BAC-Q72 mice ICV-treated with AAV9-miR-ATXN2 or AAV9-miR-Neg (control) vectors. Downregulation of *ATXN2* and *Atxn2* mRNA was evaluated by RT-dPCR (A). Downregulation of mouse and human ATXN2 proteins were evaluated using mouse-specific, human/pig/NHP and mouse/human/pig/NHP peptides; comparison performed using the Wilcoxon rank-sum test (B). Correlation expressed as coefficient of correlation (*r*) was calculated, respectively, between *ATXN2* mRNA and peptides (C) and *Atxn2* mRNA and peptides (D) were assessed using Spearman’s rank correlation. All statistical tests were two-sided, with significance defined as *p* < 0.05. Analyses were conducted using a bespoke R script.

Seven peptides, two mouse-specific, two human/pig/NHP, and three mouse/human/pig/NHP cross-reactive peptides, were tested in the protein extracted from the right brain hemispheres of mice dosed with AAV9-miR-ATXN2 or AAV9-miR-Neg vector and control naïve mice.

Of note, in a preliminary experiment, brain lysates from ICV-injected AAV9-miR-Neg vector (control) and untreated naïve mice were compared using the newly developed IP-MS method to account for any potential procedure-related effect on ATXN2 protein expression. No difference in any of the peptides was visible between naïve and AAV9-miR-Neg vector, thus confirming that surgery *per se* had no effect on the protein level and the effects noted in the AAV9-miR-ATXN2-treated animals were attributable to the treatment with the test article (Supporting Information Figure S2).

Mirroring what was seen for the RNA transcripts, a dose-dependent downregulation of both human and mouse ATXN2 proteins was shown for all seven peptides in the brain lysates tested (Figure 5B). A clear dose response in ATXN2 protein was noted for the five peptides tested belonging to the human/pig/NHP and mouse/human/pig/NHP cross-reactive groups. Although both mouse-specific peptides showed a dose–response, the magnitude of the signal across the three doses was less dramatic than that shown for the cross-reactive peptide groups, potentially attributable to the overexpression of the human ATXN2 in the transgenic animal when compared to the orthologous protein or a higher affinity of the construct tested for *ATXN2 vs Atxn2* (Figure 5B).

All peptides tested reached a statistically significant downregulation, with the exception of one mouse/human/pig/NHP cross-reactive peptide (MSSEGPPR) at the lowest dose tested (Figure 5B). A moderate positive correlation (defined as a correlation coefficient *R* between 0.3 and 0.7) is apparent between *ATXN2* mRNA downregulation and both human/pig/NHP and mouse/human/pig/NHP peptide groups (Figure 5C). Not surprisingly, no correlation was seen between *Atxn2* mRNA downregulation and mouse-specific peptides (Figure 5D), considering that only the highest dose reached significant downregulation of mouse *Atxn2* transcript.

Knockdown of *Atxn2* and *ATXN2* transcripts correlated with mouse and human protein downregulation evaluated with the selected peptides, thus demonstrating target engagement and proof of mechanism in a relevant preclinical model by AAV9-miR-ATXN2 using orthogonal methodologies.

## Discussion

The development of a drug requires multiple translational steps to ensure that the right models and biomarker strategy are in place once the lead candidate progresses beyond the research space.^14,15^ Drug development is a notoriously time-consuming and costly exercise.^16,17^ A single assay that can be used across multiple species offers an undeniable translational advantage for drug developers as it has the potential to streamline biomarker alignment, reduce redundant assay development, and shorten preclinical to clinical timelines, an efficiency gain that could significantly lower drug development costs. In this instance, cross-species translational tools have been shown to improve decision-making and reduce late-stage attrition, which is a major driver of cost in drug development.^15,18^

Target engagement is the first landmark question answered in preclinical studies, both in *in vitro* and *in vivo* models.^19^ Here, we present a strategy devised by our preclinical team to monitor target engagement following a single dose of AAV9-miR-ATXN2, an AAV9 vector carrying a miR construct designed to knock down ATXN2 with the aim of offering a disease-modifying treatment for ALS.

The IP-MS assay presented in this manuscript was designed to provide a solution that could cater to the need of discriminating between human vs murine ATXN2 protein produced by transgenic animals, while being at the same time readily translatable to analyze nonrodent model used for preclinical toxicological studies.

We started with the design of peptides specific for human ATXN2, specific for murine ATXN2, cross-reactive for human/pig/NHP, and common for mouse/human/pig/NHP.

The commercially available antibodies are cross-reactive between mouse and human ATXN2; moreover, we proved that they could recognize pig ATXN2 too.

Therefore, the optimal strategy would be to immunoprecipitate all ATXN2 proteins (human and murine) and then use the specific peptides to quantify each form as well as the total ATXN2 protein extracted from the samples. Indeed, the murine-specific peptides were designed to discriminate the endogenous murine ATXN2 protein from the human form of ATXN2 overexpressed in a BAC-Q72 transgenic mouse model.^11^

As ALS is a neurodegenerative condition associated with progressive loss of motor neurons in the brain and spinal cord, the feasibility test of the IP-MS assay was carried out on different areas of brains and spinal cords from both WT and BAC-Q72 mice. Liver samples were collected too, as ablation of ATXN2 has been linked to dysregulation of liver branched-chain amino acids and fatty acids,^20^ and therefore an organ of potential interest in transgenic animal models.

The initial step was to assess the feasibility of designing peptides that could discriminate between mouse and human ATXN2 protein. Although human-specific peptides were synthesized, our attention was focused on three classes of peptides: specific for mouse, human/pig/NHP, and mouse/human/pig/NHP cross-reactive peptides.

Our reasoning was that for a translational assay, the human specific peptides will be of little use; we therefore concentrated our efforts on the peptides that are common to human, pig, and NHP in combination with the mouse/human/pig/NHP cross-reactive ones as more translatable and suitable for the preclinical space. A different set of peptides might be tested in human biofluids; the focus of this study was to develop an assay to analyze tissues from preclinical models.

Seven peptides per group were synthesized and tested for detection in samples during the initial screening phase: three peptides for mouse, three for human/pig/NHP, and five for the mouse/human/pig/NHP cross-reactive peptide group were taken forward.

The next step was to identify the best antibody combination enabling the pull-down of the total ATXN2 (mouse and human) from the samples by testing the commercial antibodies one by one coupled with the number of peptides identified in the pull-down material.

Two antibodies were selected, based on the number of peptides identified and whether they were identified in both organs, thus decreasing the bias associated with tissue-specific variants.^21^

Spliced isoforms are conserved in mice and are present in several human tissues (brain, spinal cord, cerebellum, heart, placenta),^21–23^ albeit at different levels.^24^ The expression level of the different tissue variants might be reflected in the recovery for some of the peptides, as shown in the data presented in this study.

To prove that the assay was specific and was able to identify and measure only peptides derived from ATXN2 proteins and no other proteins accidently coprecipitated during the IP stage, brains excised from SCA2 KO and HET SCA2 mice were tested.

SCA2 KO mice are null for Atxn2 and are not transgenic for ATXN2 either, so neither mouse nor human ATXN2 protein is produced in the brains of those animals.^12^

The data presented here confirm that mouse-specific and mouse/human/pig/NHP cross-reactive peptides could be identified only in SCA2 HET mice, and no signal was detected for any of the peptides in the SCA2 KO animals.

During the design phase of the IP-MS method, we planned to use the same assay for other preclinical species, in particular Yucatan minipig and NHP (*M. mulatta*), hence, why we designed two sets of cross-reactive peptides.

In silico protein alignment vs peptides identified in the BAC-Q72 mice suggested that the antibody cocktail used to immunoprecipitate mouse and human ATXN2 should also work in Yucatan minipig samples. We therefore tested tissue lysates from neuronal and non-neuronal tissues excised from minipig using the three sets of peptides also tested in mouse tissues, mouse-specific, human/pig/NHP, and mouse/human/pig/NHP. We confirmed that the cross-reactive peptides human/pig/NHP and mouse/human/pig/NHP, but not the mouse-specific peptides, were identified in minipig tissue, further supporting that this assay can be easily translatable and used in other species to support target engagement and/or validation of ATXN2 proteins in preclinical studies.

Moreover, with the caveat that the test was conducted on a limited number of animals, our data suggest that the peptides could recognize tissue isoform of ATXN2 protein (as highlighted by the differential signals recorded in muscle vs liver vs brain/spinal cord for the different peptides).

The robustness of the methodology developed so far was also tested using a manual and an automated process, and the performances of both approaches were deemed comparable. The automated process is preferred to decrease operator-to-operator variation as well as human errors. The robustness, linearity, and reproducibility of the assay were also evaluated with positive outcomes, demonstrating that the assay is suitable for ATXN2 relative quantification.

Accuracy and %CV were evaluated in surrogate matrix using synthetic peptides and pooled tissue homogenates going through the full IP-MS workflow in triplicates. For the two human/pig/NHP peptides and the mouse/human/pig/NHP cross-reactive peptides, the accuracy and %CV showed a variation of max 20%, both in surrogate matrix and after IP-MS, with the mouse/human/pig/NHP cross-reactive peptides showing the best performance. For both mouse-specific peptides, accuracy and %CV showed a variation of max 20%; however, only one peptide (GMSPVVSEHR) showed a %CV variation of max 20% after IP-MS; the other peptide (EEIMESVLFK) was slightly higher than 20%. To note: for both peptides with higher CV, there is only one amino acid change between the mouse and the human/pig/NHP cross-reactive sequences. Specifically pertinent to this study, even though the difference was at a single amino acid level, this assay was specific enough to discriminate between mouse and other mammalian ATXN2 species.

The next step was to measure ATXN2 protein in brain samples from a Proof of Mechanism (PoM) study. A PoM study in the context of preclinical drug development has a few objectives (EUPATI, Making a medicine. Step 6: phase I-proof of mechanism, https://toolbox.eupati.eu/resources/making-a-medicine-step-6-phase-i-proof-of-mechanism/): to confirm that the test article can reach the target organ(s); specificity of interaction with the target molecule has a pharmacological effect and this effect is dose-dependent too.^25,26^

This study was designed to evaluate the ability of an AAV9 vector carrying a miRNA against *Atxn2* to infect neurons when delivered ICV and to downregulate the production of both human and mouse mRNA in brains of BAC-Q72 mice (direct target engagement). As a consequence, mouse and human ATXN2 proteins should have also been downregulated in a dose–response manner in the area treated with AAV9 test article (indirect target engagement by causing a biological response).

The data presented in this study showed that if the test article was present in the murine brains and expressed as vg (vector genome)/μg, then there was a corresponding decrease of both *ATXN2* and *Atxn2* transcripts, which in turn caused a decrease in both mouse and human ATXN2 proteins measured in the samples too.

For both mouse and human ATXN2, a dose–response was clearly demonstrated both at the protein and at the RNA level, with all peptides showing a significant downregulation even at the lowest dose tested (all but one peptide, MSSEGPPR), likely owing to the kinetics of the protein translation vs RNA transcription processes and the intrinsic stability of proteins vs RNA molecules.^27,28^ Moreover, even though the correlation coefficient presented in this study between downregulation at the mRNA and protein level is considered moderate based on statistics, it is a substantial achievement from a biological point of view, proving the fidelity of the IP-MS assay developed.

Indeed, two orthogonal methodologies that cross-validate the data collected from the PoM study is an extremely important result, thus supporting not only the mechanism of action of the drug product and objective of the study but also confidence in the assay, the data generated, and in the success of the drug product journey from its discovery to clinical development.

## Conclusion

A truly translational drug development strategy requires analytical tools that reliably perform across species, that can discriminate human targets from their orthologues, while maintaining assay platform continuity throughout the preclinical pipeline by saving costs and cutting timelines, a sore issue for drug developers. Our IP-MS assay was built and developed on the basis of these pillars. It delivers exceptional specificity, discriminating peptides that differ by a single amino acid and clearly separating human from mouse ATXN2 in BAC-Q72 transgenic tissues, which is an essential feature for studying ATXN2 overexpression and assessing target engagement. Orthogonal validation using RT-dPCR confirmed that mRNA knock-down aligns with protein knockdown, reinforcing the biological relevance of the assay despite the well-known disconnect between transcriptomic and proteomic responses. The workflow is readily translatable to other mammalian species, enabling consistent analysis across toxicology and pharmacology studies and ensuring methodological continuity across the development. More importantly, this work provides a clear blueprint for how a translational mindset, rather than a classical proteomic approach, can be applied to similar biological challenges in preclinical studies, offering a practical and adaptable strategy for species-discriminating assays in drug discovery and development.

## Supplementary Material









## Data Availability

Data sets presented in this manuscript are available within the published article and its Supporting Information. In addition, the mass spectrometry data have been made available by the authors in Panorama Public (Link to access the data: https://panoramaweb.org/ZGroD0.url).

## Notes

Peptide design and mapping using ATXN2 amino acid sequences belonging to four species: human (*H. sapiens*), mouse (*M. musculus*), minipig (*S. scrofa*), and NHP (*M. mulatta*) (PDF)
Procedural-related effects on ATXN2 protein expression in brain tissues of BAC-Q72 mice ICV treated with AAV9-miR-Neg (control) vector vs naïve (untreated) mice (PDF)
Information related to the peptide sequences designed for the study (PDF)
List of peptides taken forward for quantitation analysis (PDF)
