# Supplementary material for: Development of a Translational Assay for Identification and Quantitation of Orthologous Proteins to Aid Drug Development in Preclinical Models
Source: J Proteome Res. 2026 Sep 16;25(10):5091–102. doi: 10.1021/acs.jproteome.5c01164 (PMC13641131; doi:10.1021/acs.jproteome.5c01164)
Supplement: Supplementary file 1 [file pr5c01164_si_001.pdf]

Title: Development of a translational assay for identification and quantitation of orthologous proteins to aid drug development in pre-clinical models

Authors: Blandine Rougemont[1], Julie Borgel[1], Zoe Walker[2], Irene Zubiri[1], Ronan O'Cualain [1], Neda Ali Mohammadi Nafchi[3], Christopher E. Shaw[2,3], Ernestas Sirka[1], John Isaac[2], Carlos J. Miranda[2], Anna Ettorre[2,*]

Addresses: [1]Inoviv, Fox Court, 14 Gray's Inn Road, London WC1X 8HN, UK; [2]AviadoBio Ltd, 20 Water Street, London E14 5GX, UK; [3]Maurice Wohl Clinical Neuroscience Institute, 5 Cutcombe Road, London, SE5 9RT, UK.

SUPPORTING INFORMATION – Table of Contents:

The following supporting information is available free of charge at ACS website http://pubs.acs.org.

Figure S1 / Supplementary Figure 1: Peptide design and mapping using ATXN2 amino acid sequences belonging to four species: human (*Homo sapiens*), mouse (*Mus musculus*), minipig (*Sus scrofa*) and NHP (*Macaca mulatta*).

Figure S2 / Supplementary Figure 2: Procedural-related effects on ATXN2 protein expression in brain tissues of BAC-Q72 mice ICV treated with AAV9-miR-Neg (Control) vector *vs.* naïve (untreated) mice.

Tables S1A, S1B, S1C / Supplementary Table 1: Information related to the peptide sequences designed for the study.

Table S2 / Supplementary Table 2: List of peptides taken forward for quantitation analysis.

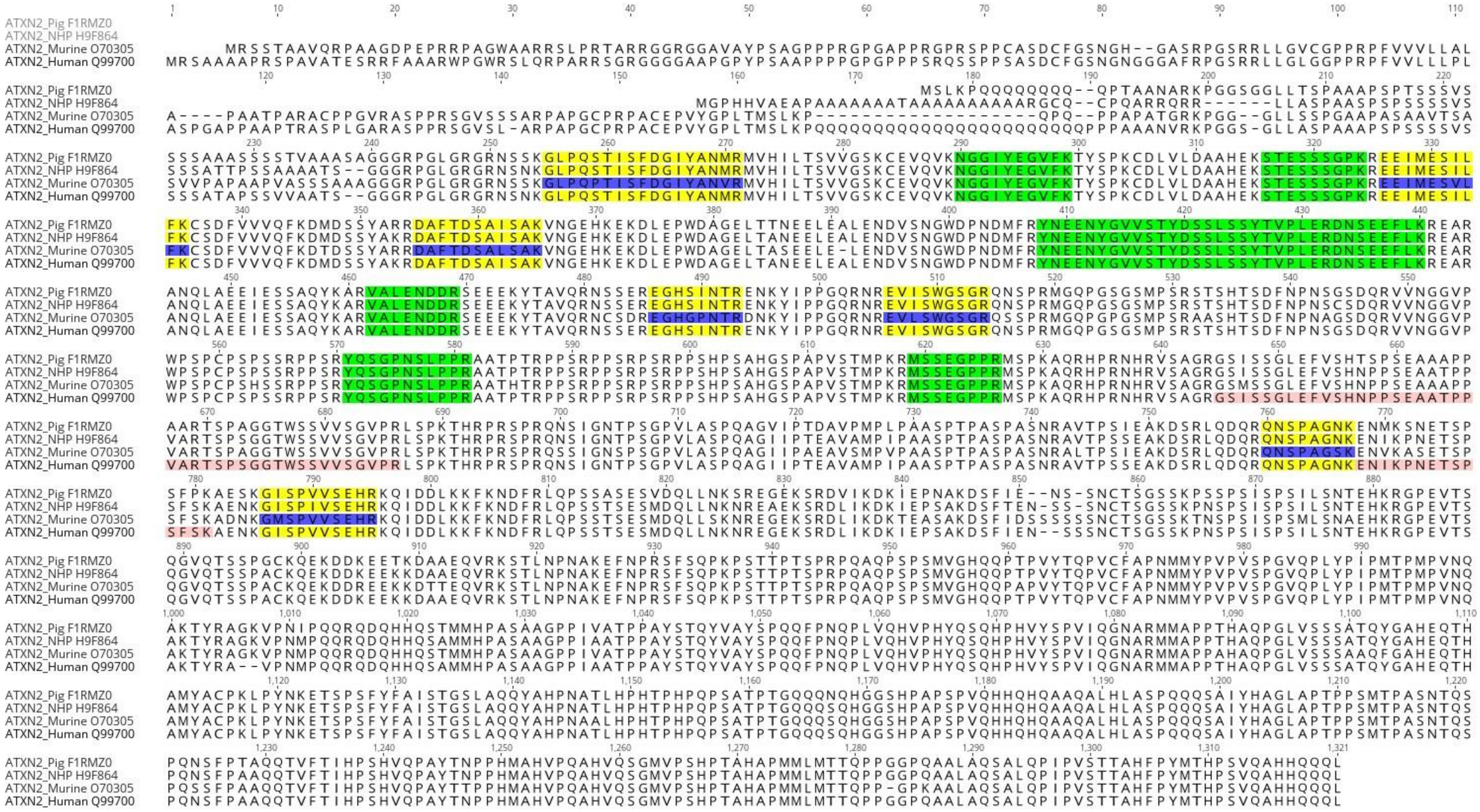

Supplementary Figure 1: Peptide design and mapping using ATXN2 amino acid sequences belonging to four species: human (*Homo sapiens*), mouse (*Mus musculus*), minipig (*Sus scrofa*) and NHP (*Macaca mulatta*). Purple: peptide specific to mouse only; pink: peptides specific to human only; yellow: peptides common to human/pig/NHP; green: cross-reactive mouse/human/pig/NHP peptides.
