# Supplementary material for: Development of a Translational Assay for Identification and Quantitation of Orthologous Proteins to Aid Drug Development in Preclinical Models
Source: J Proteome Res. 2026 Sep 16;25(10):5091–102. doi: 10.1021/acs.jproteome.5c01164 (PMC13641131; doi:10.1021/acs.jproteome.5c01164)
Supplement: Supplementary file 2 [file pr5c01164_si_002.pdf]

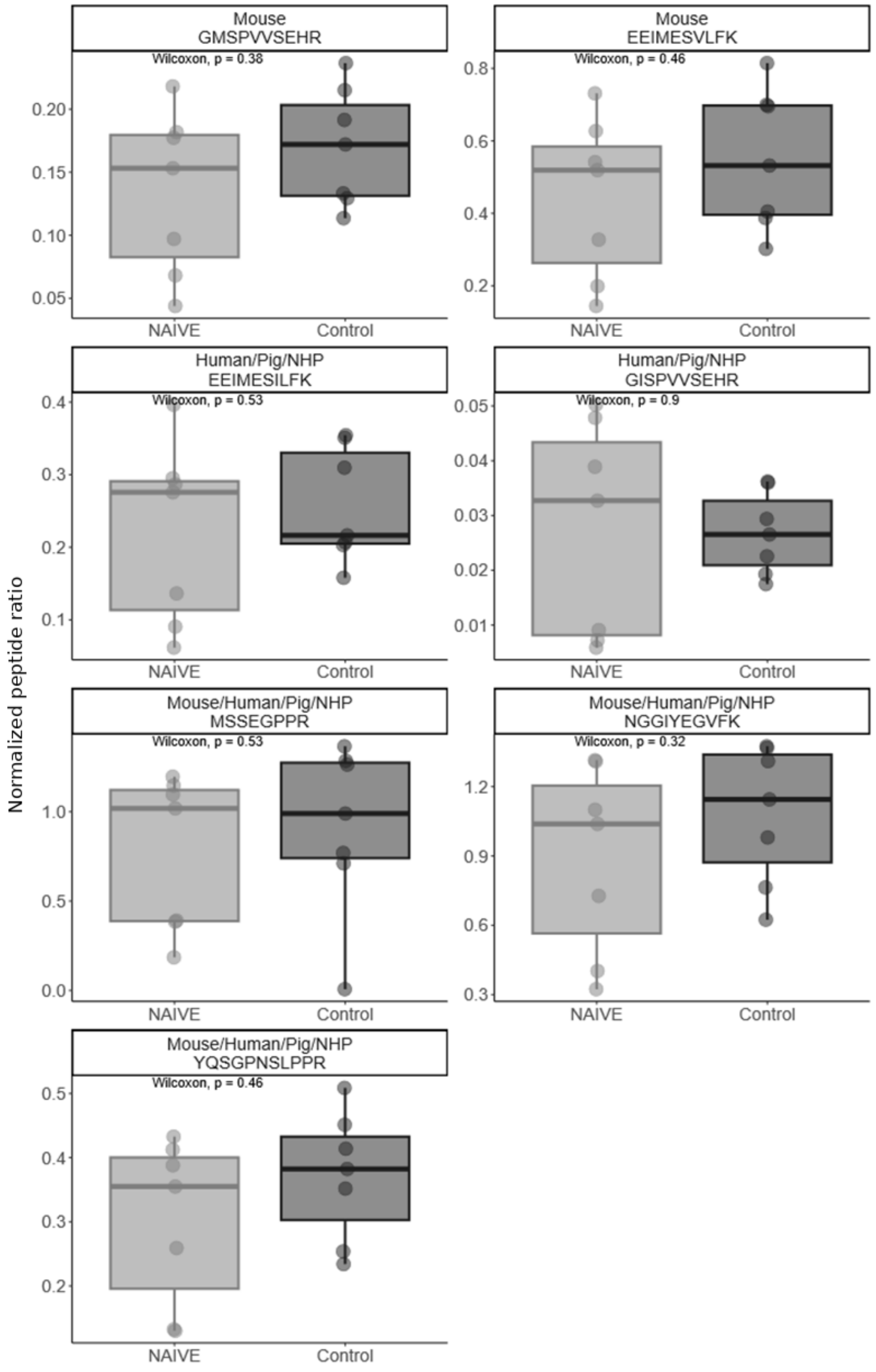

Supplementary Figure 2: Procedural-related effects on ATXN2 protein production was assessed in brain tissue excised from BAC-Q72 mice ICV treated with AAV9-miR-Neg (Control) vector vs. naïve (untreated) mice. The cortex from the right hemisphere was snap frozen for protein extraction. Mouse-specific, human/pig/NHP and mouse/human/pig/NHP cross-reactive peptides were used to quantify down-regulation of mouse and human ATXN2 proteins. Naïve and Control samples were compared to prove that no effect on ATXN2 protein level was due to ICV injection for all peptides tested. Wilcoxon rank-sum test was used to assess difference between ICV-injected and non- injected groups and p value is reported within each graph of the figure. All statistical tests were two-sided, with significance defined as $p < 0.05$. Analyses were conducted using a bespoke R script.
