# Supplementary material for: Development of a Translational Assay for Identification and Quantitation of Orthologous Proteins to Aid Drug Development in Preclinical Models
Source: J Proteome Res. 2026 Sep 16;25(10):5091–102. doi: 10.1021/acs.jproteome.5c01164 (PMC13641131; doi:10.1021/acs.jproteome.5c01164)
Supplement: Supplementary file 3 [file pr5c01164_si_003.pdf]

## Supplementary Table 1- Information related to the peptide sequence designed for the study.

### Supplementary Table 1A- List of peptides, groups and sequences

| Specificity | Peptide Sequence | Comments | Used for analysis |
|---|---|---|---|
| Human/Pig/NHP | DAFTDSAISAK | Isobaric with one mouse peptide | Monitored only |
| Human/Pig/NHP | EVISWGSGR | Isobaric with one mouse peptide | Monitored only |
| Human/Pig/NHP | EGHSINTR | Detected in samples | Yes |
| Human/Pig/NHP | QNSPAGNK | Not detected in samples | Discarded |
| Human/Pig/NHP | EEIMESILFK | Detected in samples | Yes |
| Human/Pig/NHP | GISPVVSEHR | Detected in samples | Yes |
| Human/Pig/NHP | GLPQSTISFDGIYANMR | Not soluble | Discarded |
| Mouse/Human/Pig/NHP | NGGIYEGVFK | Detected in samples | Yes |
| Mouse/Human/Pig/NHP | DNSEEFLK | Detected in samples | Yes |
| Mouse/Human/Pig/NHP | VALENDDR | Detected in samples | Yes |
| Mouse/Human/Pig/NHP | MSSEGPPR | Detected in samples | Yes |
| Mouse/Human/Pig/NHP | YQSGPNSLPPR | Detected in samples | Yes |
| Mouse/Human/Pig/NHP | YNEENYGVVSTYDSSLSSYTVPLER | Not soluble | Discarded |
| Mouse/Human/Pig/NHP | STESSSGPK | Bad LC profile | Discarded |
| Mouse | DAFTDSALSAK | Isobaric with one Human/Pig/NHP peptide | Monitored only |
| Mouse | GLPQPTISFDGIYANVR | Detected in samples | Yes |
| Mouse | EVLSWGSGR | Isobaric with one Human/Pig/NHP peptide | Monitored only |
| Mouse | EGHGPNTR | Bad LC profile | Discarded |
| Mouse | EEIMESVLFK | Detected in samples | Yes |
| Mouse | QNSPAGSK | Bad LC profile | Discarded |
| Mouse | GMSPVVSEHR | Detected in samples | Yes |
| Human | ENIKPNETSPSFSK | Not tested in samples | Not used in the study |
| Human | TSPSGGTWSSVVSGVPR | Not tested in samples | Not used in the study |
| Human | GSISSGLEFVSHNPPSEAATPPVAR | Not tested in samples | Not used in the study |

*N.B.: 28 peptides were originally designed, 4 Human specific peptides couldn't be synthetized due to high hydrophobicity or sequence length.*

## Supplementary Table 1B – Optimised Transition Parameters

| ATXN2 | Peptide sequence | Peptide type | Q1 | Q3 | RT (min) | Cone Voltage (V) | Collision Energy (V) |
|---|---|---|---|---|---|---|---|
| Mouse/Human/Pig/NHP | MSSEGPPR | Endogenous | 430.8 | 426.2 | 4.5 | 31 | 22 |
| Mouse/Human/Pig/NHP | MSSEGPPR | Endogenous | 430.8 | 642.3 | 4.5 | 31 | 20 |
| Mouse/Human/Pig/NHP | MSSEGPPR | Endogenous | 430.8 | 729.3 | 4.5 | 31 | 20 |
| Mouse/Human/Pig/NHP | MSSEGPPR | Heavy SIL | 435.8 | 436.2 | 4.5 | 31 | 22 |
| Mouse/Human/Pig/NHP | MSSEGPPR | Heavy SIL | 435.8 | 652.3 | 4.5 | 31 | 20 |
| Mouse/Human/Pig/NHP | MSSEGPPR | Heavy SIL | 435.8 | 739.35 | 4.5 | 31 | 20 |
| Human/Pig/NHP | EGHSINTR | Endogenous | 457.3 | 355.1 | 2 | 47 | 22 |
| Human/Pig/NHP | EGHSINTR | Endogenous | 457.3 | 364.2 | 2 | 47 | 18 |
| Human/Pig/NHP | EGHSINTR | Endogenous | 457.3 | 503.2 | 2 | 47 | 22 |
| Human/Pig/NHP | EGHSINTR | Endogenous | 457.3 | 590.2 | 2 | 47 | 22 |
| Human/Pig/NHP | EGHSINTR | Heavy SIL | 462.2 | 360.7 | 2 | 47 | 22 |
| Human/Pig/NHP | EGHSINTR | Heavy SIL | 462.2 | 369.2 | 2 | 47 | 18 |
| Human/Pig/NHP | EGHSINTR | Heavy SIL | 462.2 | 513.3 | 2 | 47 | 22 |
| Human/Pig/NHP | EGHSINTR | Heavy SIL | 462.2 | 600.3 | 2 | 47 | 22 |
| Mouse/Human/Pig/NHP | VALENDDR | Endogenous | 466.3 | 290.1 | 5.1 | 31 | 26 |
| Mouse/Human/Pig/NHP | VALENDDR | Endogenous | 466.3 | 381.1 | 5.1 | 31 | 14 |
| Mouse/Human/Pig/NHP | VALENDDR | Endogenous | 466.3 | 761.3 | 5.1 | 31 | 20 |

| | | | | | | | |
|---|---|---|---|---|---|---|---|
| Mouse/Human/Pig/NHP | VALENDDR | Heavy SIL | 471.3 | 300.0 | 5.1 | 31 | 26 |
| Mouse/Human/Pig/NHP | VALENDDR | Heavy SIL | 471.3 | 386.1 | 5.1 | 31 | 14 |
| Mouse/Human/Pig/NHP | VALENDDR | Heavy SIL | 471.3 | 771.3 | 5.1 | 31 | 20 |
| Mouse/Human/Pig/NHP | DNSEEFLK | Endogenous | 491.4 | 260.1 | 6.7 | 33 | 24 |
| Mouse/Human/Pig/NHP | DNSEEFLK | Endogenous | 491.4 | 536.3 | 6.7 | 33 | 22 |
| Mouse/Human/Pig/NHP | DNSEEFLK | Endogenous | 491.4 | 752.3 | 6.7 | 33 | 22 |
| Mouse/Human/Pig/NHP | DNSEEFLK | Heavy SIL | 495.4 | 268.1 | 6.7 | 33 | 24 |
| Mouse/Human/Pig/NHP | DNSEEFLK | Heavy SIL | 495.4 | 544.3 | 6.7 | 33 | 22 |
| Mouse/Human/Pig/NHP | DNSEEFLK | Heavy SIL | 495.4 | 760.3 | 6.7 | 33 | 22 |
| Mouse (*) | EVL/ISWGSGR | Endogenous | 495.8 | 376.1 | 7 | 41 | 14 |
| Mouse (*) | EVL/ISWGSGR | Endogenous | 495.8 | 762.2 | 7 | 41 | 18 |
| Mouse (*) | EVL/ISWGSGR | Endogenous | 495.8 | 649.2 | 7 | 41 | 20 |
| Mouse (*) | EVL/ISWGSGR | Heavy SIL | 500.8 | 386.2 | 7 | 41 | 14 |
| Mouse (*) | EVL/ISWGSGR | Heavy SIL | 500.8 | 772.3 | 7 | 41 | 18 |
| Mouse (*) | EVL/ISWGSGR | Heavy SIL | 500.8 | 659.3 | 7 | 41 | 20 |
| Human | ENIKPNETSPSFSK | Endogenous | 526.5 | 283.1 | 5.7 | 40 | 20 |
| Human | ENIKPNETSPSFSK | Endogenous | 526.5 | 565.2 | 5.7 | 40 | 20 |
| Human | ENIKPNETSPSFSK | Endogenous | 526.5 | 652.3 | 5.7 | 40 | 20 |
| Human | ENIKPNETSPSFSK | Heavy SIL | 529.5 | 287.1 | 5.7 | 40 | 20 |
| Human | ENIKPNETSPSFSK | Heavy SIL | 529.5 | 573.2 | 5.7 | 40 | 20 |
| Human | ENIKPNETSPSFSK | Heavy SIL | 529.5 | 660.3 | 5.7 | 40 | 20 |
| Human/Pig/NHP | GISPVVSEHR | Endogenous | 540.8 | 412.1 | 5.7 | 35 | 20 |
| Human/Pig/NHP | GISPVVSEHR | Endogenous | 540.8 | 455.6 | 5.7 | 35 | 18 |
| Human/Pig/NHP | GISPVVSEHR | Endogenous | 540.8 | 627.2 | 5.7 | 35 | 26 |
| Human/Pig/NHP | GISPVVSEHR | Heavy SIL | 545.8 | 417.3 | 5.7 | 35 | 20 |
| Human/Pig/NHP | GISPVVSEHR | Heavy SIL | 545.8 | 460.7 | 5.7 | 35 | 18 |
| Human/Pig/NHP | GISPVVSEHR | Heavy SIL | 545.8 | 637.3 | 5.7 | 35 | 26 |

| | | | | | | | |
|---|---|---|---|---|---|---|---|
| Mouse/Human/Pig/NHP | NGGIYEGVFK | Endogenous | 542.5 | 294.1 | 7.6 | 35 | 24 |
| Mouse/Human/Pig/NHP | NGGIYEGVFK | Endogenous | 542.5 | 342.1 | 7.6 | 35 | 14 |
| Mouse/Human/Pig/NHP | NGGIYEGVFK | Endogenous | 542.5 | 742.3 | 7.6 | 35 | 14 |
| Mouse/Human/Pig/NHP | NGGIYEGVFK | Heavy SIL | 546.5 | 302.1 | 7.6 | 35 | 24 |
| Mouse/Human/Pig/NHP | NGGIYEGVFK | Heavy SIL | 546.5 | 342.1 | 7.6 | 35 | 14 |
| Mouse/Human/Pig/NHP | NGGIYEGVFK | Heavy SIL | 546.5 | 750.3 | 7.6 | 35 | 14 |
| Mouse | GMSPVVSEHR | Endogenous | 549.8 | 412.2 | 5.4 | 47 | 18 |
| Mouse | GMSPVVSEHR | Endogenous | 549.8 | 455.7 | 5.4 | 47 | 18 |
| Mouse | GMSPVVSEHR | Endogenous | 549.8 | 627.3 | 5.4 | 47 | 26 |
| Mouse | GMSPVVSEHR | Heavy SIL | 554.8 | 417.2 | 5.4 | 47 | 18 |
| Mouse | GMSPVVSEHR | Heavy SIL | 554.8 | 460.7 | 5.4 | 47 | 18 |
| Mouse | GMSPVVSEHR | Heavy SIL | 554.8 | 637.32 | 5.4 | 47 | 26 |
| Human/Pig/NHP (*) | DAFTDSAI/LSAK | Endogenous | 563.3 | 470.1 | 6.8 | 41 | 16 |
| Human/Pig/NHP (*) | DAFTDSAI/LSAK | Endogenous | 563.3 | 691.2 | 6.8 | 41 | 20 |
| Human/Pig/NHP (*) | DAFTDSAI/LSAK | Endogenous | 563.3 | 792.3 | 6.8 | 41 | 20 |
| Human/Pig/NHP (*) | DAFTDSAI/LSAK | Heavy SIL | 567.3 | 474.3 | 6.8 | 41 | 16 |
| Human/Pig/NHP (*) | DAFTDSAI/LSAK | Heavy SIL | 567.3 | 699.4 | 6.8 | 41 | 20 |
| Human/Pig/NHP (*) | DAFTDSAI/LSAK | Heavy SIL | 567.3 | 800.4 | 6.8 | 41 | 20 |
| Mouse/Human/Pig/NHP | YQSGPNSLPPR | Endogenous | 608.5 | 292.1 | 5.9 | 33 | 20 |
| Mouse/Human/Pig/NHP | YQSGPNSLPPR | Endogenous | 608.5 | 369.2 | 5.9 | 33 | 20 |
| Mouse/Human/Pig/NHP | YQSGPNSLPPR | Endogenous | 608.5 | 924.4 | 5.9 | 33 | 20 |
| Mouse/Human/Pig/NHP | YQSGPNSLPPR | Heavy SIL | 613.5 | 292.1 | 5.9 | 33 | 20 |
| Mouse/Human/Pig/NHP | YQSGPNSLPPR | Heavy SIL | 613.5 | 379.2 | 5.9 | 33 | 20 |
| Mouse/Human/Pig/NHP | YQSGPNSLPPR | Heavy SIL | 613.5 | 934.4 | 5.9 | 33 | 20 |
| Mouse | EEIMESVLFK | Endogenous | 613.0 | 259.0 | 9 | 43 | 22 |
| Mouse | EEIMESVLFK | Endogenous | 613.0 | 593.3 | 9 | 43 | 16 |
| Mouse | EEIMESVLFK | Endogenous | 613.0 | 722.4 | 9 | 43 | 18 |

| | | | | | | | |
|---|---|---|---|---|---|---|---|
| Mouse | EEIMESVLFK | Heavy SIL | 616.8 | 259.0 | 9 | 43 | 22 |
| Mouse | EEIMESVLFK | Heavy SIL | 616.8 | 601.3 | 9 | 43 | 16 |
| Mouse | EEIMESVLFK | Heavy SIL | 616.8 | 730.4 | 9 | 43 | 18 |
| Human/Pig/NHP | EEIMESILFK | Endogenous | 620.0 | 294.1 | 9.5 | 50 | 26 |
| Human/Pig/NHP | EEIMESILFK | Endogenous | 620.0 | 736.4 | 9.5 | 50 | 24 |
| Human/Pig/NHP | EEIMESILFK | Endogenous | 620.0 | 867.4 | 9.5 | 50 | 22 |
| Human/Pig/NHP | EEIMESILFK | Heavy SIL | 623.8 | 302.2 | 9.5 | 50 | 26 |
| Human/Pig/NHP | EEIMESILFK | Heavy SIL | 623.8 | 744.4 | 9.5 | 50 | 24 |
| Human/Pig/NHP | EEIMESILFK | Heavy SIL | 623.8 | 875.5 | 9.5 | 50 | 22 |
| Human | TSPSGGTWSSVVSGVPR | Endogenous | 831.1 | 272.1 | 8 | 49 | 26 |
| Human | TSPSGGTWSSVVSGVPR | Endogenous | 831.1 | 515.3 | 8 | 49 | 26 |
| Human | TSPSGGTWSSVVSGVPR | Endogenous | 831.1 | 736.9 | 8 | 49 | 26 |
| Human | TSPSGGTWSSVVSGVPR | Heavy SIL | 835.9 | 282.1 | 8 | 49 | 26 |
| Human | TSPSGGTWSSVVSGVPR | Heavy SIL | 835.9 | 525.3 | 8 | 49 | 26 |
| Human | TSPSGGTWSSVVSGVPR | Heavy SIL | 835.9 | 741.9 | 8 | 49 | 26 |
| Human | GSISSGLEFVSHNPPSEAATPPVAR | Endogenous | 836.7 | 539.3 | 8 | 47 | 26 |
| Human | GSISSGLEFVSHNPPSEAATPPVAR | Endogenous | 836.7 | 596.9 | 8 | 47 | 24 |
| Human | GSISSGLEFVSHNPPSEAATPPVAR | Endogenous | 836.7 | 640.3 | 8 | 47 | 22 |
| Human | GSISSGLEFVSHNPPSEAATPPVAR | Heavy SIL | 839.7 | 549.3 | 8 | 47 | 26 |
| Human | GSISSGLEFVSHNPPSEAATPPVAR | Heavy SIL | 839.7 | 601.9 | 8 | 47 | 24 |
| Human | GSISSGLEFVSHNPPSEAATPPVAR | Heavy SIL | 839.7 | 650.3 | 8 | 47 | 22 |
| Mouse | GLPQPTISFDGIYANVR | Endogenous | 924.7 | 726.9 | 9.5 | 50 | 32 |
| Mouse | GLPQPTISFDGIYANVR | Endogenous | 924.7 | 792.3 | 9.5 | 50 | 36 |
| Mouse | GLPQPTISFDGIYANVR | Endogenous | 924.7 | 839.5 | 9.5 | 50 | 30 |
| Mouse | GLPQPTISFDGIYANVR | Heavy SIL | 929.7 | 731.9 | 9.5 | 50 | 32 |
| Mouse | GLPQPTISFDGIYANVR | Heavy SIL | 929.7 | 802.3 | 9.5 | 50 | 36 |
| Mouse | GLPQPTISFDGIYANVR | Heavy SIL | 929.7 | 844.5 | 9.5 | 50 | 30 |

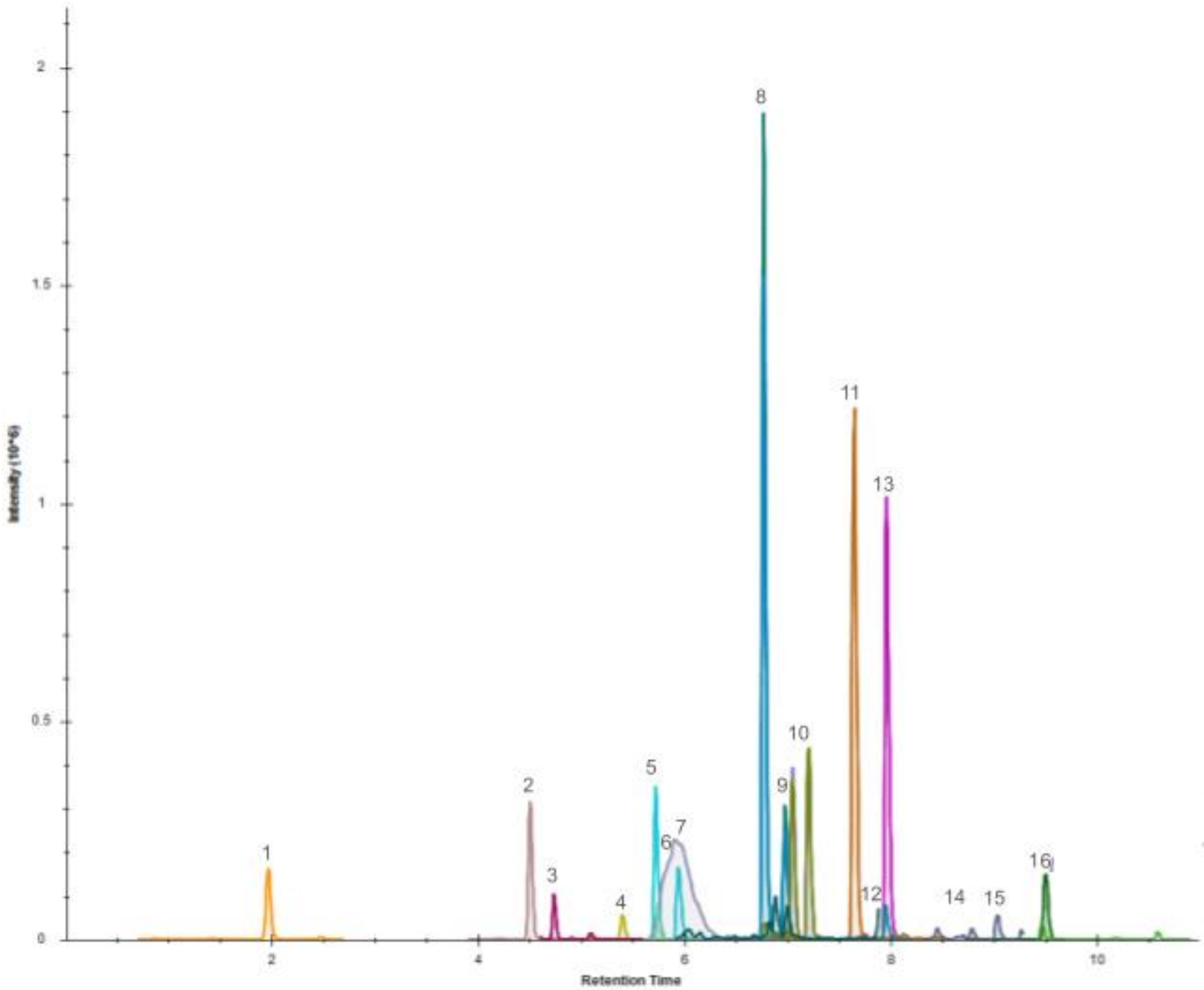


1. EGHSINTR
2. MSSEGPPR
3. VALENDDR
4. GMSPVVSEHR
5. GISPVVSEHR
6. ENIKPNETSPSFSK
7. YQSGPNSLPPR
8. DAFTDSAI/LSAK
9. DNSEEFLK
10. EVI/LSWGSGR
11. NGGIYEGVFK
12. GSISSGLEFVSHNPPSEAATPPVAR
13. TSPSGGTWSSVVSGVPR
14. EEIMESIVFK
15. EEIMESILFK
16. GLPQPTISFDGIYANVR

**Supplementary Table 1: Information related to the peptides designed for the study.** (**A**) List of peptides designed; groups belonging to human-specific peptides; mouse-specific peptides; human/pig/NHP peptides; mouse/human/pig/NHP peptides); performance assessment during the initial phase. (**B**) Optimised transition parameters associated with each peptide including Q1 (Precursor m/z), Q3 (Fragment m/z), retention time (RT, expressed in min); Cone Voltage (V) and Collision Energy (V). Peptides marked with (*) are isobaric peptides not used for quantitation. (**C**) Chromatograms exemplifying RT, peak intensity and shape. N.B. The transition table in Supplementary Table 1B corresponds to the exact masses monitored on our instrument. These values were obtained during optimization upon tuning each peptide in Intellistart (Waters). This process can result in a deviation of the theoretical value of <0.5 Da, as Intellistart records the apex of the measured mass, thus explaining the small difference observed in the transition list uploaded in Panorama, which displays only theoretical values.
