# Supplementary material for: Development of a Translational Assay for Identification and Quantitation of Orthologous Proteins to Aid Drug Development in Preclinical Models
Source: J Proteome Res. 2026 Sep 16;25(10):5091–102. doi: 10.1021/acs.jproteome.5c01164 (PMC13641131; doi:10.1021/acs.jproteome.5c01164)
Supplement: Supplementary file 4 [file pr5c01164_si_004.pdf]

Specificity	Peptide Sequence	CV%	Used for analysis
Human/ Pig/ NHP	EGHSINTR	43.5	Not to be used
Human/ Pig/ NHP	EEIMESILFK	14.3	Selected for quantification
Human/ Pig/ NHP	GISPVVSEHR	18.2	Selected for quantification
Mouse/ Human/ Pig/ NHP	NGGIYEGVFK	4.9	Selected for quantification
Mouse/ Human/ Pig/ NHP	DNSEEFLK	26.2	Monitored, not used for quantification
Mouse/ Human/ Pig/ NHP	VALENDDR	58.5	Not to be used
Mouse/ Human/ Pig/ NHP	MSSEGPPR	5.8	Peptide prone to carry-over keep as back-up
Mouse/ Human/ Pig/ NHP	YQSGPNSLPPR	8.0	Selected for quantification
Mouse	GLPQPTISFDGIYANVR	81.1	Not to be used
Mouse	EEIMESVLFK	23.4	Selected for quantification
Mouse	GMSPVVSEHR	16.2	Selected for quantification

Supplementary Table 2: List of peptides taken forward for quantitation analysis.

IP-MS was performed in triplicate on tissue lysates from a pooled BAC-Q72 cortex lysate. Most of the monitored peptides displayed a %CV of <25%.

For future relative quantification data analysis, optimal peptides from each ATXN2 peptide group specificity will be selected.
